# Niobium Doping in Pt/C Electrocatalysts: Synthesis and Catalytic Activity for Methanol Oxidation

**DOI:** 10.1021/acsomega.5c11822

**Published:** 2026-04-24

**Authors:** Leonardo Alexandre Veltrone, José Antônio Salgado Garizado, Yunier Garcia-Basabe, Josimar Ribeiro, Diogo M. F. Santos, José Javier Sáez Acuña, José Ricardo Cezar Salgado

**Affiliations:** † Latin American Institute of Technology, Infrastructure and Territory, Federal University of Latin American Integration, Foz do Iguaçu, Paraná 85867-970, Brazil; ‡ Latin American Institute of Life Sciences and Nature, Federal University of Latin American Integration, Foz do Iguaçu, Paraná 85867-970, Brazil; § Laboratory of Research and Development in Electrochemistry, Department of Chemistry, Federal University of Espírito Santo, Vitória, Espírito Santo 29075-910, Brazil; ∥ Center of Physics and Engineering of Advanced Materials, Laboratory of Physics for Materials and Emerging Technologies, Chemical Engineering Department, Instituto Superior Técnico, Universidade de Lisboa, Lisbon 1049-001, Portugal; ⊥ Center for Natural and Human Sciences, Federal University of ABC, São Paulo 09210-580, Brazil

## Abstract

Fuel cells powered by renewable sources are gaining prominence for their high energy conversion efficiency and low pollutant emissions. These electrochemical devices convert chemical energy directly into electrical energy and utilize sustainable fuels, such as hydrogen and low-molecular-weight alcohols. However, challenges related to catalyst cost, efficiency, and carbon monoxide (CO) poisoning hinder the widespread adoption of alcohol fuel cells. This study investigates the role of niobium (Nb) in the catalytic performance of platinum (Pt)-based anodes. A PtNb/C electrocatalyst in a 3:1 ratio is synthesized by sodium borohydride reduction, followed by physical and electrochemical characterizations. The Pt_3_Nb_1_/C catalyst exhibits a lower onset potential (0.45 V) than commercial Pt/C (0.55 V), demonstrating superior electrocatalytic activity for the methanol oxidation reaction and improved CO tolerance. Structural analysis reveals that Nb is present as oxides (Nb_*x*_O_*y*_), which induce electronic interactions with Pt, facilitating CO oxidation. At the electrochemically active area (63.2 m^2^ g^–1^ Pt_3_Nb_1_/C vs 51.8 m^2^ g^–1^ Pt/C), the Nb-doped catalyst demonstrates higher Pt utilization efficiency and oxidation current densities. These findings highlight the potential of Nb-based materials to enhance the performance and durability of Pt catalysts in fuel cell applications, contributing to the advancement of sustainable energy technologies.

## Graphical abstract

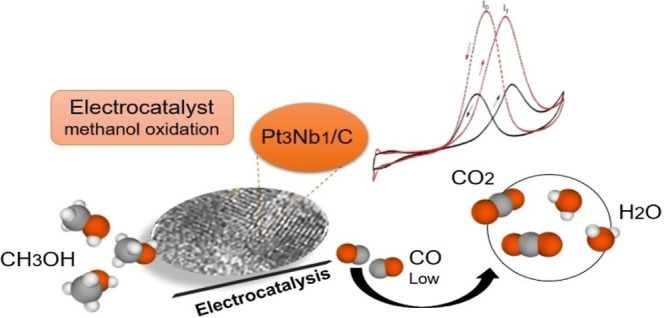

## 1 Introduction

Fuel cells convert chemical energy into electrical energy using a set of electrodes (anode and cathode, where the oxidation and reduction reactions occur, respectively), an electrolyte (which allows the ionic transport between the electrodes), the electrocatalysts (which accelerate the electrochemical reactions), and bipolar plates (which distribute the fuel and oxidant gases and are electrical conductors). To improve the performance of alcohol fuel cells, it is necessary to use efficient anode catalysts. Among them, platinum (Pt) exhibits high catalytic activity in acidic media; however, it is a noble metal with a high cost and limited supply.^1,2^ Furthermore, Pt-based catalysts are easily poisoned by carbon monoxide (CO), which decreases their efficiency and leads to the formation of undesirable intermediates.^1,3^ For these reasons, fuel cell research is shifting toward the use of alternative metals to replace or combine with Pt in the anode. A primary strategy to enhance CO tolerance of Pt-based electrocatalysts involves alloying Pt with a second, more oxophilic element.^4^ This approach has led to the investigation of bimetallic and trimetallic catalysts, such as Pt-M and Pt-M1M2 (M, M1, or M2 = Pd, Te, Co, Rh, Sn, and Ru), supported on different matrices, which aim to reduce the amount of noble metal needed and increase catalytic efficiency.^5–11^ The synergistic interaction in these alloys often facilitates the oxidation of CO at lower potentials, a mechanism crucial for catalyst durability.^4,12^ Recent advances in electrocatalysis have highlighted the importance of surface chemistry and low-dimensional catalyst architectures for improving the methanol oxidation reaction (MOR). Strategies such as engineering metal oxide interfaces, tailoring nanoparticle morphology, and developing low-dimensional or single-atom catalysts have been widely explored to enhance catalytic activity, CO tolerance, and stability. These approaches rely on electronic structure modulation, optimized adsorption of reaction intermediates, and synergistic effects at the catalyst surface. Consequently, a wide variety of Pt-based nanostructured catalysts and hybrid materials have been proposed as promising candidates for direct methanol fuel cells (DMFCs) and related electrochemical energy conversion technologies.^13–15^

The Pt_3_M atomic ratio in bimetallic electrocatalysts has shown exceptional promise for optimizing performance in the MOR. This 3:1 composition effectively balances electronic modification and alloy stability, enhancing both catalytic activity and tolerance to poisoning intermediates. Density functional theory (DFT) studies, such as those by Du et al.,^16^ have elucidated reaction pathways on Pt_3_Ni­(100) surfaces, revealing favorable kinetics for O–H bond cleavage and dual dehydrogenation routes leading to CO formation. These insights underscore the mechanistic advantages of Pt_3_M surfaces. Experimental works further support these findings: Chen et al.^17^ demonstrated that intermetallic ordering in core–shell Pt_3_M@Pt structures enhances MOR activity via strain-induced modulation of CO adsorption. Similarly, Chen et al.^18^ developed Pt_3_Cu alloys on nanoporous WO_3_, achieving superior mass and specific activities through lattice compression effects. Peng et al.^19^ reported high-performance Pt_3_Mn nanowire networks with improved durability and activity. Most recently, Yu et al.^20^ synthesized ordered Pt_3_Ti and Pt_3_V nanoparticles with exceptional mass activities, emphasizing the crucial role of atomic ordering and tailored synthesis. Collectively, these advances highlight the Pt_3_M composition as a highly effective design strategy for next-generation MOR electrocatalysts in DMFCs.

In this context, niobium (Nb) metal is a promising material for addressing these challenges. In Brazil, approximately 90% of Nb production is converted into ferroniobium alloys containing about 60–70% Nb, primarily used in high-strength low-alloy steels. The remaining 10% is allocated to specialized applications, including the Nb oxides and other high-value products.^21^ The Nb atom and its oxides present numerous potential applications in the field of electrocatalysis. Recent research has advanced toward the structural modification of catalysts, either by adjusting the metal-to-oxide mass ratio or by optimizing the support material used to anchor metals and oxides. A significant frontier in this area is the development of heterostructured catalysts, in which the interface between different materials, such as Pt and metal oxides, creates unique active sites that enhance catalytic performance.^22,23^ In their experiments, Ramesh et al.^24^ demonstrated that the NbPt_3_ nanoparticles exhibit greater electrochemical stability than the pure Pt nanoparticles when subjected to repeated cycling in sulfuric acid (H_2_SO_4_) solution. Additionally, these nanoparticles show enhanced catalytic activity in the electrooxidation of ethanol and/or formic acid. Further improvements in activity and CO tolerance have been observed with the development of bimetallic PtNi/NbN-C catalysts for methanol electrooxidation in alkaline media.^25^ These findings highlight the combined interaction between the metallic components and the Nb-based support, which plays a critical role in enhancing both the durability and the electrocatalytic performance of the system.^25^ Recent studies have also investigated Nb-doped Pt electrocatalysts to elucidate the origin of enhanced activity and CO tolerance in the MOR. For instance, Zhang et al.^26^ reported that Nb doping can modify the electronic structure of Pt nanoparticles and promote the removal of poisoning intermediates during MOR. However, studies addressing Nb-modified Pt nanoparticles supported on carbon and their interaction with Nb-derived oxide species under acidic electrochemical conditions remain relatively limited.

In recent decades, niobium oxides (Nb_*x*_O_*y*_) have gained importance in the high-tech industry due to their specialized industrial applications, particularly in catalytic processes, with a focus on heterogeneous catalysis and photocatalysis.^27^ Justin et al.^28^ showed that the performance of methanol electrooxidation depends on the amount of niobium pentoxide (Nb_2_O_5_) loading, and peak current densities from methanol electrooxidation are significantly higher (∼80%) in Pt–Nb_2_O_5_(2:2)/C than in Pt–Ru(2:1)/C. Furthermore, the Pt–Nb_2_O_5_(2:2)/C electrocatalyst exhibited a lower current density decay with time than Pt–Ru(2:1)/C, suggesting good tolerance to CO-like intermediates. The enhanced electrode response is attributed to the combined interaction between Pt and Nb_2_O_5_. A modern catalyst design that promotes strong metal-support interactions (SMSI) favors the formation of interfacial Pt–O–metal bonds, facilitates electron transfer at the catalytic interface, and consequently results in superior performance in the MOR.^29^ Rocha et al.^30^ assessed the electrocatalytic activity of Pt–Nb_2_O_5_ nanoparticles for ethanol oxidation. They found that crystalline Pt–Nb_2_O_5_/C generated higher currents than the amorphous counterpart. However, it was not effective at completely oxidizing ethanol, yielding acetic acid as the primary product. In this sense, greater efficiency in the complete oxidation of ethanol was achieved with the deposited catalyst. In another study, the authors also found that Nb presence has a noticeable effect on catalyst CO tolerance, as evidenced by a reduction in the CO removal initiation potential. Additionally, the CO/O_2_ polarization curves show a decrease in current density in the presence of Nb. Chun et al.^31^ synthesized a CO-tolerant Nb_2_O_5_-promoted Pt/C catalyst for DMFCs. The authors found that the Pt–Nb_2_O_5_/C catalyst thermally treated at 400 °C presented the best electrocatalytic activity for CO and methanol oxidation. The authors suggested the good performance stemmed from a combined interaction between Pt and Nb_2_O_5._ ^31^ Beyond catalyst composition, the support material is crucial for electrocatalytic performance. Although conventional carbon blacks such as Vulcan XC-72 are widely used, innovative supports have been explored to improve dispersion, stability, and electronic properties. In this context, Li et al.^32^ developed Mo_2_C-based materials exhibiting d-band characteristics similar to those of platinum, enabling Mo_2_C to act effectively as a support or cocatalyst in direct methanol fuel cells. Moreover, its unique hexagonal crystalline structure, particularly when combined with nitrogen-doped carbon, contributes to enhanced overall catalytic performance.^32^ Shi et al.^33^ studied Pt nanorods supported on Nb-doped ceria as a promising anode catalyst for polymer electrolyte fuel cells, deliberately avoiding carbon-based supports due to their susceptibility to electrochemical corrosion. This catalyst system also contributes to enhanced polymer electrolyte membrane stability, primarily attributed to reduced oxidative degradation. The anisotropic morphology of the Pt nanorods, in combination with electronic and structural interactions involving cerium (Ce) and Nb species, underpins the observed improvements in both catalytic activity and electrochemical stability for the hydrogen oxidation reaction.^33^ Furthermore, incorporating Nb oxide coatings onto electrocatalytic surfaces has been shown to further augment the durability of nanostructured catalysts, while simultaneously enhancing catalytic performance and mitigating the adverse effects of impurity poisoning.^34^ These advanced strategies are part of a broader effort in catalysis that extends to maximizing metal utilization, with single-atom catalysts representing the ultimate frontier in efficiency, where every metal atom is an active site.^35^

The present work reports the synthesis and characterization of bimetallic Pt_3_Nb_1_ catalysts supported on Vulcan carbon (Pt_3_Nb_1_/C), prepared via a chemical reduction method using sodium borohydride (NaBH_4_) as the reducing agent. This study aims to investigate the structural, morphological, and electrochemical effects of Nb incorporation into the Pt matrix, considering its ability to alter the electronic structure of Pt, enhance nanoparticle dispersion, and improve resistance to CO poisoning. In addition to these catalytic advantages, Nb is a strategically relevant material, particularly for Brazil, which accounts for approximately 90% of global supply. The novelty of this work lies in using a specific Pt–Nb atomic ratio (3:1) combined with a NaBH_4_-based reduction route, an approach not extensively explored in the literature, which offers potential for improved catalytic activity and durability. This synthesis strategy is expected to influence the material’s physicochemical properties and its electrochemical behavior during MOR. Therefore, Pt–Nb-based catalysts emerge as a promising alternative to reduce noble-metal loading while improving catalytic efficiency and long-term durability in low-temperature fuel cell systems.

## 2 Experimental Section

### 2.1 Catalyst Preparation

Platinum–niobium (Pt–Nb) was deposited on carbon (20 wt % metal) by chemical reduction with borohydride. Required quantities of Vulcan XC-72R carbon black (Cabot Corp.) were added to isopropyl alcohol and Milli-Q water (1:1), followed by an ultrasonic bath for 15 min. In a standard procedure, adequate amounts of hexachloroplatinum acid (H_2_PtCl_6_·6H_2_O, Aldrich) and niobium pentachloride (NbCl_5_, Sigma-Aldrich) were dissolved in the mixture. The solution was homogenized under magnetic stirring for 10 min. Next, chemical reduction was carried out using sodium borohydride (NaBH_4_) (0.1 mol L^–1^, pH 13), which was slowly added to the reaction vessel using a buret (30 drops per minute). NaBH_4_ is widely employed as a strong reducing agent in the synthesis of nanoparticles due to its ability to promote multielectron transfer. In alkaline media, the complete oxidation of the BH_4_^–^ ion can release up to eight electrons per ion, as shown in eq 1.

1 $${BH}_{4}^{-}+{8OH}^{-}\to {BO}_{2}^{-}+{6H}_{2}O+{8e}^{-}{,E}^{{}^{\circ}}=-1.24\;V\;vs.RHE$$

This behavior is particularly observed in direct borohydride fuel cells (DBFCs), which highlight the high reducing potential of the BH_4_^–^ ion.^36^ However, electrochemical studies demonstrate that complete oxidation of BH_4_^–^ does not always occur, particularly on platinum (Pt)-based catalysts.^37,38^ Considering these aspects, the synthesis of the Pt_3_Nb_1_/C catalyst was carefully conducted to ensure efficient incorporation of Nb and removal of byproducts. The system was kept agitated to ensure complete reduction. The catalyst slurry was then vacuum-filtered and washed repeatedly with Milli-Q (Millipore) water to remove any byproducts. Finally, the catalyst was dried in an oven for 24 h at 60 °C and finely ground. Henceforth, this sample will be referred to as Pt_3_Nb_1_/C. Commercial Pt/C from Sigma-Aldrich was employed as a reference material for comparative morphological, physical, and electrochemical characterizations against the synthesized Pt_3_Nb_1_/C catalyst.

### 2.2 Physical and Morphological Characterization

The physical structure of the catalysts was analyzed using high-resolution transmission electron microscopy (HRTEM) with a Thermo Fischer Talos F200X-G2 microscope (acceleration voltage: up to 200 kV Field Emission Gun electron).

The metal content was evaluated by subjecting the samples to thermal treatment in an inert atmosphere (N_2_/Ar) at 900 °C for 1 h with oxygen flow (50 mL min^–1^) and heating rate of 10 °C min^–1^ (PerkinElmer STA8000 equipment). This process burns off the carbon support, leaving only the metal mass.

X-ray diffraction (XRD) patterns were recorded using a PANalytical Empyrean multipurpose X-ray system with Cu Kα radiation (λ = 1.54184 Å) in the angular range of 20° to 90° (2θ) to determine the presence of crystalline phases and estimate the average crystal size.

X-ray photoelectron spectroscopy (XPS) analysis was carried out using a Thermo Scientific K-Alpha spectrometer equipped with an Al Kα source (1486.6 eV) as the excitation energy. The XPS spectra were processed using the Casa XPS software package (version 2.3.2), employing pseudo-Voigt profile functions, which are linear combinations of Gaussian (*G*) and Lorentzian (*L*) functions. Background correction was performed using a Shirley function. The C 1s photoemission peak (C–C) at a binding energy of 284.5 eV was used to monitor potential surface charging effects (i.e., shifts in electron energy).

### 2.3 Electrochemical Characterization

The catalysts were electrochemically characterized by cyclic voltammetry (CV) and chronoamperometry (CA). A catalyst suspension (2 mg) in 500 μL of H_2_O with 20 μL of Nafion was prepared and dispersed in an ultrasonic bath for 40 min. Using an automatic pipet, the suspension was carefully deposited onto the working electrode until it reached a volume of 10 μL. After drying, the electrode was inserted into a three-electrode glass cell containing 0.5 mol L^–1^ H_2_SO_4_ solution. A reversible hydrogen electrode (RHE) reference was prepared by performing electrolysis on the same supporting electrolyte. For the CV experiments, the system was first purged with nitrogen for 20 min and then cycled between 50 and 1200 mV at 20 mV s^–1^. Then, CV and CA measurements were used to determine the catalytic activity for the MOR. To this end, the corresponding amount of methanol was added to the same supporting electrolyte to obtain a 0.5 mol L^–1^ solution. The system was cycled at 20 mV s^–1^ between 50 and 1200 mV several times until a stable response was obtained. The current density was reported by normalizing the current to the geometric surface area of the glassy carbon electrode (0.07 cm^–2^). CA experiments were carried out using the same reaction medium as in the case of CV. In this case, the system was polarized at an anode potential of 0.55 V, and the current was monitored for 30 min. All electrochemical measurements were performed at a constant temperature of 25.0 ± 0.1 °C. The results were normalized with respect to the electrochemically active area. The platinum loading on the electrode was 0.052 mg_Pt_ cm^–2^ for the Pt/C catalyst and 0.072 mg_Pt_ cm^–2^ for the Pt_3_Nb_1_/C catalyst.

## 3 Results and Discussion

### 3.1 X-ray Diffraction (XRD)

XRD analysis was carried out to investigate possible structural modifications induced by Nb incorporation in the Pt/C system. The resulting patterns for the commercial Pt/C and Pt_3_Nb_1_/C catalysts are presented in Figure 1.

**Figure 1:**
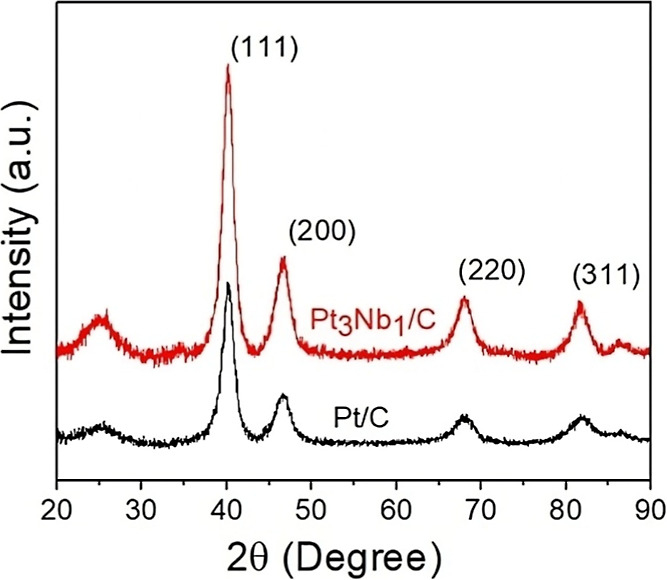
XRD patterns of commercial Pt/C and Pt_3_Nb_1_/C.

Typical peaks associated with the Pt FCC crystal structure (ICDD Card: 04-0802) can be observed at 40°, 46°, 68°, and 82°, corresponding to reflections with Miller indices (111), (200), (220), and (311), respectively. No peaks associated with any Nb crystalline phase are present, and no shift of the Bragg peaks was detected. No significant changes were observed in this parameter, regardless of the presence of Nb. Consequently, Nb is expected to be present as an amorphous oxide phase. According to the literature, the most likely state under the synthesis conditions (aqueous medium) is Nb_2_O_5_, resulting from the hydrolysis of NbCl_5_.^39^

The absence of peaks related to the crystalline form of niobium oxide (Nb_2_O_5_) in the XRD analyses of the Pt_3_Nb_1_ system anchored on Vulcan carbon can be attributed to several structural and synthetic factors. First, the low concentration of Nb in the material, combined with its high dispersion, hinders the formation of crystalline domains detectable by XRD. Furthermore, the calcination temperatures typically used in supported catalytic systems (generally below 400 °C) are insufficient to induce the crystallization of Nb_2_O_5_, which requires temperatures above 700 °C for the formation of orthorhombic or pseudohexagonal phases.^40^

The interaction with the carbon support may promote the stabilization of noncrystalline species, suppressing the formation of well-defined crystalline phases. Under these conditions, advanced techniques such as TEM and XPS are particularly suitable for detecting the presence and characterizing the oxidation state of Nb, even in the absence of crystalline phases detectable by conventional methods.^41^

The Scherrer equation^42^ (eq 2) was used to calculate the average crystallite size (*d*, nm), assuming a spherical shape

2 d=Kλ/βcos⁡θ

where *K* is the shape factor (commonly 0.9), λ is the wavelength of the X-ray radiation used (e.g., 1.5406 Å for Cu Kα), β is the full width at half-maximum of the peak (in radians), and θ is the Bragg diffraction angle (in radians).

Subsequently, the specific surface area (*S*, m^2^ g_Pt_^–1^) can be estimated from eq 3 using the average crystallite size (*d*, nm) obtained from Scherrer’s eq (eq 2)­

3 $$S=6\times 10^{3}/\left(d\rho \right)$$

where ρ is the material density in g cm^–3^.

The parameters estimated for each catalyst from eqs 2 and 3, along with their metal content, are presented in Table 1.

**Table 1:** Metal Content, Average Particle Diameter (*d*), Material Density (ρ), and Specific Surface Area (*S*)

catalysts	metal content (wt %)	*d*_XRD_ (nm)	ρ (g cm^–3^)	*S* (m^2^ g_Pt_^–1^)
Pt/C	20	3.6 ± 0.2	21.5	78
Pt_3_Nb_1_/C	28	4.2 ± 0.2	15.6	92

Comparing the data between Pt/C and Pt_3_Nb_1_/C catalysts (Table 1) reveals significant differences in their physicochemical properties. The Pt_3_Nb_1_/C catalyst shows a higher metal content (28 wt %) than Pt/C (20 wt %), which can favor a greater density of catalytically active sites. Despite the larger crystallite size observed for Pt_3_Nb_1_/C (4.2 nm vs 3.6 nm for Pt/C), this material exhibits a higher specific surface area (92 m^2^ g_Pt_^–1^) compared to Pt/C (78 m^2^ g_Pt_^–1^). This increase in specific surface area suggests that Nb in the Pt–Nb alloy not only alters the structural properties but can also induce electronic modifications in Pt, thereby increasing the availability of active sites for electrochemical reactions. The observed differences in crystallite size and electrochemical surface area between the Pt/C and Pt_3_Nb_1_/C catalysts may be attributed to variations in synthesis parameters, including the nature of the metal precursors, the rate of addition of reducing agents, the purity of the starting materials, and the reduction temperature.^43^ These factors can significantly influence particle nucleation and growth, ultimately affecting the final particle size.

### 3.2 X-ray Photoelectron Spectroscopy (XPS)

To investigate the role of Nb incorporation in modifying the surface chemistry and electronic environment of Pt, a comparative XPS analysis of the Pt/C and Pt_3_Nb_1_/C samples was carried out. The surface chemical composition of the Pt/C and Pt_3_Nb_1_/C samples was determined from the XPS survey spectra shown in Figure 2a. All expected elements for each analyzed sample (C, O, Pt, and Nb) were detected. The atomic percentage (at. %) of each element in the samples was determined from quantitative analysis of the survey spectra and is summarized in Table 2. An atomic Pt/Nb ratio of 3/1 was observed for the Pt_3_Nb_1_/C sample, which closely matches the molar ratio used in the sample preparation procedure.

**Figure 2:**
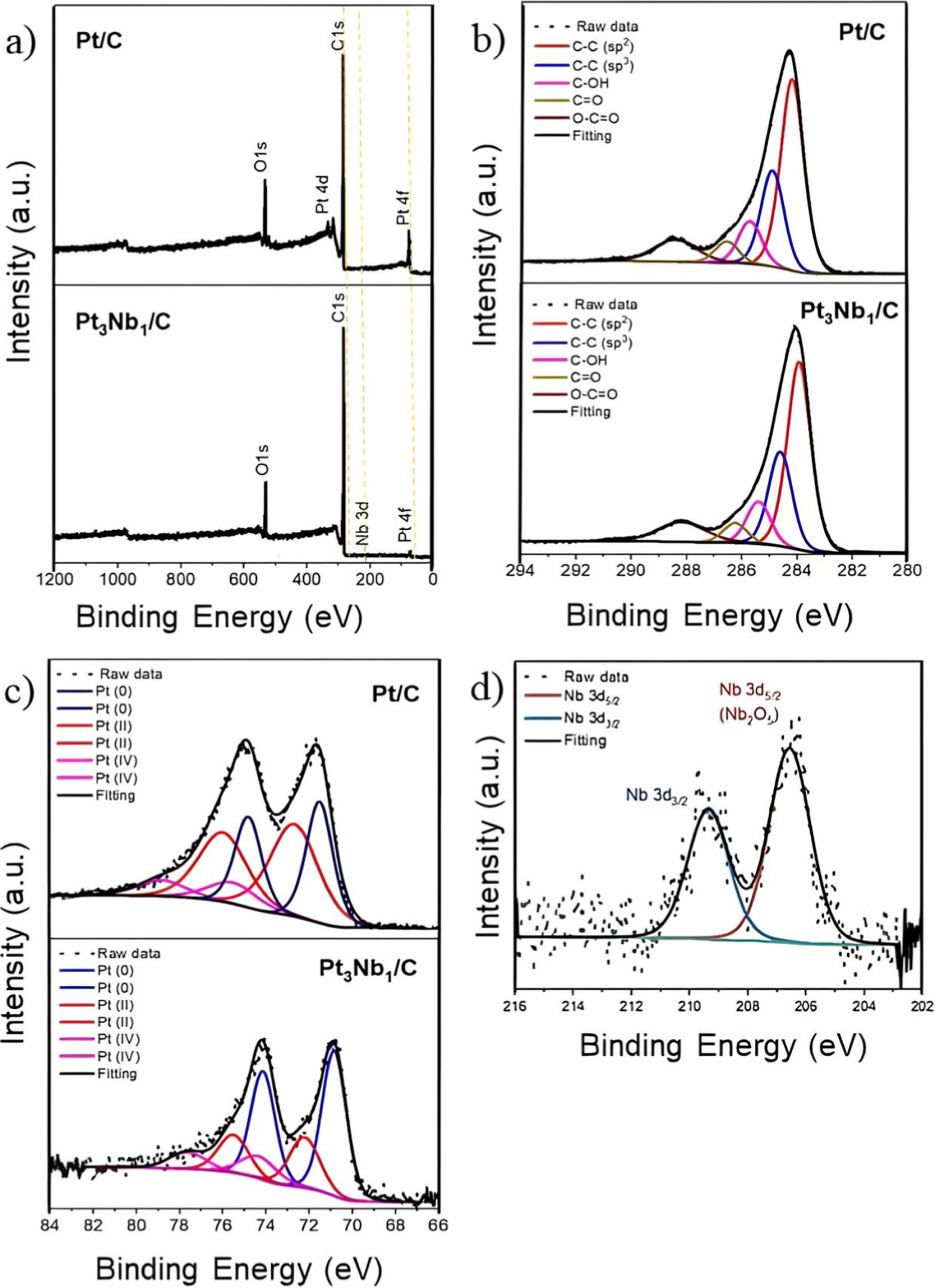
(a) XPS survey spectra showing peaks assigned to the transition of the main elements of Pt/C and Pt_3_Nb_1_/C, (b) C 1s XPS spectra fitting of Pt/C and Pt_3_Nb_1_/C, (c) Pt 4f XPS spectra of Pt/C and Pt_3_Nb_1_/C, and (d) Nb 3d XPS spectra of Pt_3_Nb_1_/C.

**Table 2:** Atomic Composition of the Sample as Determined from Quantitative Analysis of Survey Spectra

catalysts	C (at. %)	O (at. %)	Pt (at. %)	Nb (at. %)
Pt/C	84.6	11.4	4.0	-
Pt_3_Nb_1_/C	89.0	9.8	0.89	0.31

Figure 2b shows the XPS C 1s spectra of Pt/C and Pt_3_Nb_1_/C. The spectra were deconvoluted into components corresponding to sp^2^-hybridized carbon at 283.9 eV, sp^3^-hybridized carbon at 285.0 eV, C–OH bonding at 286.1 eV, C=O bonding at 286.6 eV, and O=C–OH bonding at 288.4 eV, values that are consistent with those reported in the literature.^44^ The main fitting parameters for the XPS C 1s spectra are presented in Table 3. No significant differences were observed between the spectra of the Pt/C and Pt_3_Nb_1_/C samples. Both samples exhibited a high concentration of oxygen-containing functional groups (see Table 3), suggesting concurrent carbon oxidation during the electrochemical process. This oxidation mechanism effectively describes the XPS results of the synthesized products.

**Table 3:** Results of C 1s XPS Spectra Fitting

	C–C (sp^2^)		C–C (sp^3^)		C–OH		C=O		O–C=O	
catalysts	BE (eV)	at. %	BE (eV)	at. %	BE (eV)	at. %	BE (eV)	at. %	BE (eV)	at. %
Pt/C	284.1	48.8	284.9	24.4	285.7	10.9	286.5	5.5	288.4	10.4
Pt_3_Nb_1_/C	283.9	48.8	284.6	24.5	285.4	11.0	286.2	5.5	288.2	10.6

The XPS Pt 4f spectra of Pt/C and Pt_3_Nb_1_/C are shown in Figure 2c. These spectra are characterized by three Pt species. The spin–orbit doublet of metallic Pt (0) species appeared at binding energies of 71.4 and 74.8 eV, corresponding to Pt 4f_7/2_ and Pt 4f_5/2_, respectively. Pt 4f_7/2_ and Pt 4f_5/2_ correspond to Pt­(II) oxidized species appearing at binding energies (BE) of 72.7 and 72.4 eV, respectively. On the other hand, Pt 4f_7/2_ and Pt 4f_5/2_ associated with Pt­(IV) oxide are localized at BE 75.5 and 74.2 eV, respectively.^45^ The main parameters obtained from a quantitative analysis of these spectra are summarized in Table 4. The presence of oxidized Pt species, Pt­(II) and Pt­(IV), may be responsible for the incomplete reduction of Pt species.^46^ A significant reduction by approximately 50% of Pt­(II) species was observed for the Pt_3_Nb_1_/C sample when compared to the Pt/C. Moreover, the Pt 4f peaks of PtNb/C are shifted toward lower binding energy values. These changes indicate that Nb incorporation alters the electronic environment of Pt. The suppression of Pt­(II) suggests that Nb stabilizes Pt in its metallic state, thereby hindering surface oxidation. The shift to lower binding energies is consistent with an increased electron density around Pt atoms, which can be rationalized by electronic modulation induced by Nb incorporation, given Nb’s more electropositive character. This Nb–Pt electronic interaction enriches the electron density at Pt sites and modifies its local electronic configuration. Similar electronic interaction effects have been predicted by DFT studies for intermetallic Nb–Pt compounds as well as for Pt supported on Nb-containing oxides, where the presence of Nb species modifies the Pt d-band center and contributes to the stabilization of the metallic state.^47–49^ Such charge redistribution is expected to directly affect the catalytic properties. By tuning the electronic state of Pt, Nb incorporation can modulate the adsorption strength of reaction intermediates, particularly relevant for electrocatalytic processes.

**Table 4:** Results of Pt 4f XPS Spectra Fitting

	Pt (0)		Pt (II)		Pt (IV)	
catalysts	BE (eV)	at. %	BE (eV)	at. %	BE (eV)	at. %
Pt/C	71.5	37.1	72.7	51.2	75.5	11.7
Pt_3_Nb_1_/C	70.8	58.7	72.4	26.9	74.2	14.4

To determine the oxidation state and chemical environment of Nb in the Pt_3_Nb_1_/C sample, the Nb 3d XPS region was carefully deconvoluted and fitted, as shown in Figure 2d. The spectrum was resolved into a single spin–orbit doublet, confirming the presence of only one Nb chemical environment. The two fitted components located at approximately 207.4 and 210.2 eV are assigned to the Nb 3d_5/2_ and Nb 3d_3/2_ levels, respectively. The spin–orbit splitting of approximately 2.8 eV confirms the expected doublet structure of Nb 3d. The oxidation state assignment is primarily based on the Nb 3d_5/2_ binding energy (∼207.4 eV), which matches well with reported values for Nb^5+^ species in Nb_2_O_5_. No additional components at lower binding energies, typically associated with Nb^4+^ or Nb^3+^ species, were required to achieve a satisfactory fit.^50^ The fitted intensity ratio between the Nb 3d_5/2_ and Nb 3d_3/2_ components (3:2), together with the full width at half-maximum (fwhm) values in the range of 1.1–1.2 eV, further supports the assignment to a single, well-defined Nb^5+^ species with good spectral resolution.

### 3.3 High-Resolution Transmission Electron Microscopy (HRTEM)

High-resolution transmission electron microscopy (HRTEM) and selected area electron diffraction (SAED) analyses provide comprehensive structural and morphological insights into the Pt/C and Pt_3_Nb_1_/C catalysts, as shown in Figure 3. The Pt/C sample displays well-dispersed nanoparticles with narrow size distribution and high sphericity on the carbon support, suggesting a uniform nucleation process with an average particle size of 1.88 ± 0.20 nm (distribution histogram insert Figure 3a). HRTEM images reveal well-defined lattice fringes with a characteristic interplanar spacing of approximately 0.224 nm, which consistently appears and disappears across the scanned regions (Figure 3b). This dynamic visibility under electron-beam navigation indicates a specific zone-axis orientation and confirms the presence of a preferential crystallographic plane, identified as the (111) plane of face-centered cubic (fcc) Pt.^51,52^ In contrast, the Pt_3_Nb_1_/C sample shows notable differences. Although the nanoparticles remain well-dispersed, a broader size distribution (average particle size of 3.10 ± 0.38 nm) and slightly irregular morphology are observed (Figure 3d). The results indicate that the addition of Nb atoms modifies the nucleation and growth mechanisms during synthesis, where Nb can expand the Pt lattice spacing, producing a stress effect.^53^ The HRTEM analysis (Figure 3e) reveals lattice fringes with interplanar spacings of 0.289 nm, which are less regular and slightly strained compared to those of the Pt/C sample. This slight expansion can be ascribed to interfacial interactions between Pt and highly dispersed Nb_2_O_5_ species, which impose local lattice strain without forming long-range crystalline Nb phases detectable by XRD, as discussed in Section 3.1. It should be noted that HRTEM probes local atomic-scale distortions, whereas XRD reflects the long-range, ensemble-averaged crystal structure; therefore, localized strain effects, particularly considering the small proportion of Nb incorporated, may be averaged out and not result in a measurable Bragg peak shift in conventional XRD measurements. The Pt 4f XPS spectra further support this interpretation, as the observed shift toward lower binding energies indicates electronic modification of Pt induced by Nb at the interface. Similar interfacial effects have been reported for Pt in contact with Nb_2_O_5_, where reduced Nb centers promote charge transfer, and for Pt supported on Nb-doped oxides such as TiO_2_, where electron donation to Pt modifies both the Pt 4f binding energy and catalytic activity.^54,55^ Figure 3c,f present the SAED patterns of the Pt/C and Pt_3_Nb_1_/C catalysts, respectively. The SAED pattern of Pt_3_Nb_1_/C (Figure 3f) reveals the coexistence of crystalline and amorphous phases. The diffraction rings can be indexed to the (111), (200), and (220) planes of face-centered cubic Pt, while the presence of a broad diffuse ring indicates amorphous or highly disordered regions, likely associated with Nb incorporation into the Pt lattice. In contrast, the SAED pattern of Pt/C (Figure 3c) predominantly reflects the crystalline nature of Pt nanoparticles.

**Figure 3:**
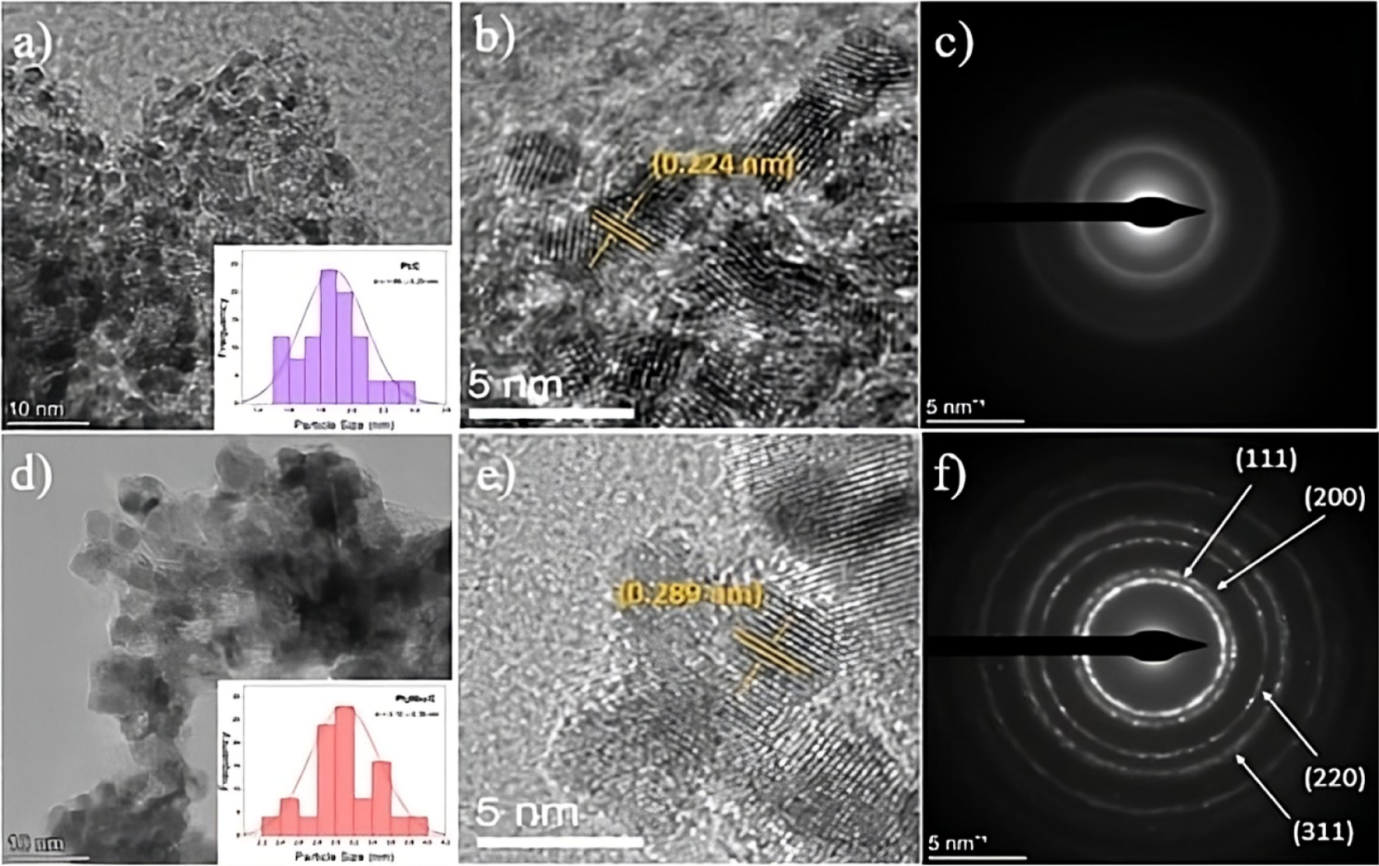
(a) HRTEM image of Pt/C with the histogram in the inset, (b) HRTEM image of Pt/C showing the interplanar spacing, (c) SAED pattern of Pt/C, (d) HRTEM image of Pt_3_Nb_1_/C with the histogram in the inset, (e) HRTEM image of Pt_3_Nb_1_/C showing the interplanar spacing, and (f) SAED pattern of Pt_3_Nb_1_/C.

### 3.4 Cyclic Voltammetry (CV)

In the CVs of the commercial Pt/C and Pt_3_Nb_1_/C electrocatalysts (Figure 4), the adsorption processes of atomic hydrogen (H_ads_) occur between potentials of 0.050 and 0.30 V vs RHE. This hydrogen is formed by the reduction of H^+^ ions present in the 0.5 mol L^–1^ H_2_SO_4_ solution (negative-going scan) and the oxidation of the adsorbed hydrogen (positive-going scan). This process is reversible because the charges involved in both processes are identical, and no shifts are observed between the peak maxima of hydrogen adsorption and oxidation as the scan rate increases. In this potential region, the voltametric behavior is highly sensitive to crystallographic orientation, as surfaces with different atomic packing arrangements exhibit distinct hydrogen adsorption energies.^56^

**Figure 4:**
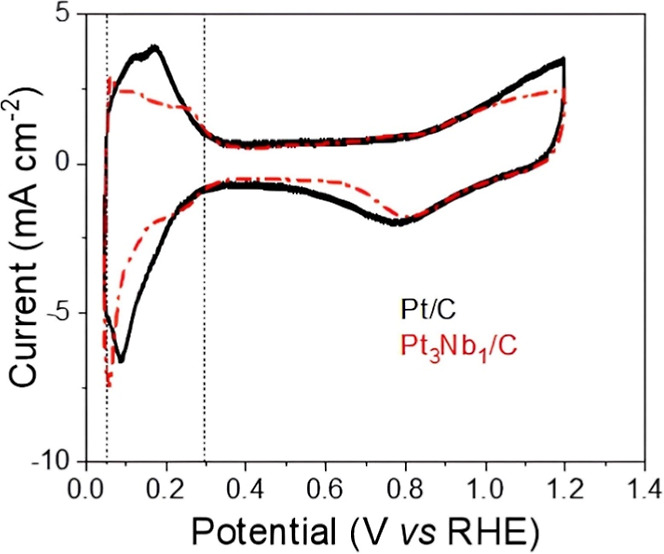
Cyclic voltammograms (catalyst area) for commercial Pt/C (20 wt % Pt) and Pt_3_Nb_1_/C electrocatalysts at 20 mV s^–1^, in 0.5 mol L^–1^ H_2_SO_4_ at 25 °C.

The electrochemically active area of the catalysts was 51.8 m^2^ g^–1^ for the Pt/C catalyst and 63.2 m^2^ g^–1^ for the Pt_3_Nb_1_/C catalyst. These values were obtained by integrating the charge in the hydrogen adsorption region of the CV in H_2_SO_4_, assuming 210 μC cm^–2^ as the oxidation charge for a monolayer of H_2_ on a smooth Pt surface.^42^ Comparing the electrochemically active area calculated from CV with the specific area obtained from particle size (XRD), it is observed that the active area value was higher in the Nb-doped material. These results indicate that a significant fraction of the Pt particles present in the electrode is effectively used in the electrochemical process. Some variations in active area measurements can be attributed to the limited exposed Pt area and/or the surface area of the material in contact with the electrolyte in thin-film porous electrodes.^43^ CV studies were performed in the presence of methanol (Figure 5a). Two well-defined peaks were observed in the forward and reverse scans for the catalysts, namely *I*_f_ (forward peak, on the right) and *I*_b_ (backward peak, on the left). The higher current ratio (*I*_f_/*I*_b_) demonstrates a better catalytic performance. The ratio of the current peaks (*I*_f_ and *I*_b_) observed between the electrocatalysts corresponds to 1.11 for Pt/C and 0.94 for Pt_3_Nb_1_/C. This is because a higher ratio corresponds to a smaller *I*_b_ peak, implying that fewer CO molecules are adsorbed on the catalyst and, consequently, a lower CO oxidation rate.^57^ The Pt_3_Nb_1_/C catalyst exhibited a significantly higher peak current density (∼36 mA cm^–2^) than that of Pt/C (∼16 mA cm^–2^) during MOR. It was noted that the oxidation current density of methanol was within the potential range of 0.45 to 0.85 V vs RHE. For potentials above 0.85 V, in the oxide formation region, a decrease in oxidation current density was observed. In the study by Chung et al.,^58^ the authors proposed that the decrease in current density above 0.85 V is due to the formation of oxygenated species on Pt surface atoms, which inhibit MOR.

**Figure 5:**
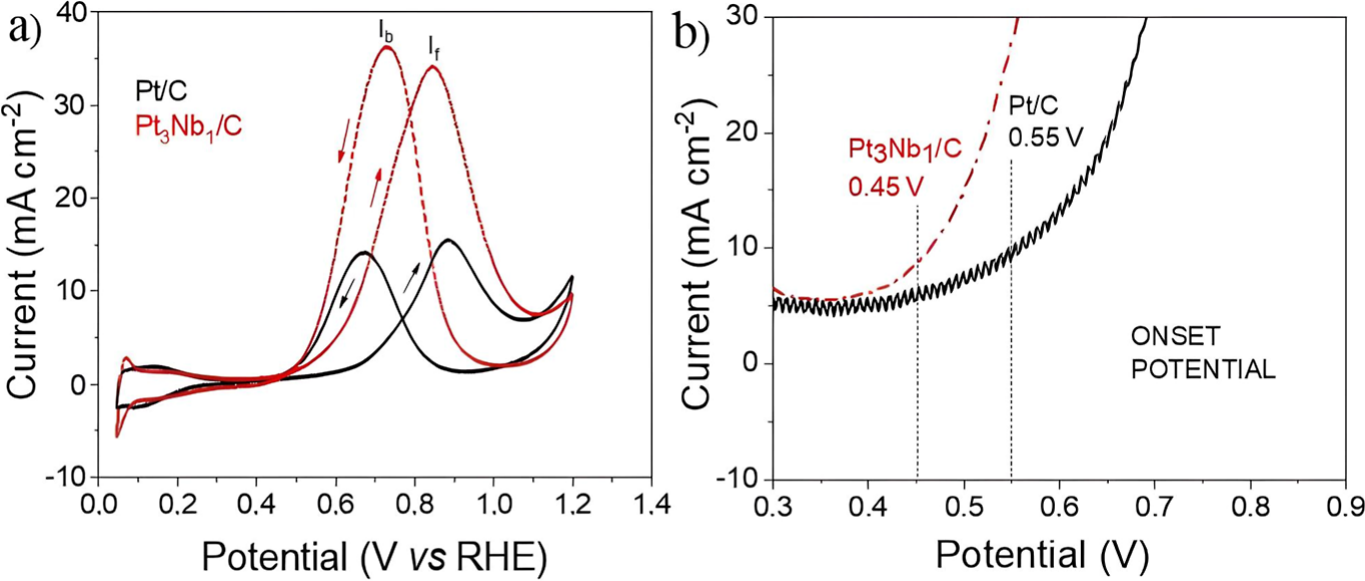
(a) Cyclic voltammograms (catalyst area) in the presence of methanol for the commercial Pt/C (20 wt % Pt) and Pt_3_Nb_1_/C electrocatalysts at 20 mV s^–1^ in 0.5 M H_2_SO_4_ + 0.5 M CH_3_OH at 25 °C and (b) the onset potentials for MOR for the same electrocatalysts.

The oxidation of methanol during the positive scan was a key factor in evaluating the catalyst activity, yielding an onset potential of 0.55 V for commercial Pt/C and 0.45 V for Pt_3_Nb_1_/C (Figure 5b).

It is well-known that CO, an intermediate in MOR, strongly adsorbs onto Pt, blocking the active site for MOR and dramatically decreasing efficiency. The decrease in the onset potential may be related to a strong electronic effect between Pt and Nb_*x*_O_*y*_, which weakens the CO adsorption strength on Pt sites. As previously discussed, the Pt_3_Nb_1_/C electrocatalyst interacts with niobium oxides (Nb_2_O_5_-amorphous), a factor that, through the combined catalytic effect, may contribute to improved catalytic performance and reduced CO poisoning. As reported in the literature,^59–61^ for reducible oxides, the CO adsorbed on the Pt surface reacts with oxygen species released onto the Pt sites from the oxide surface. After donating its surface oxygen to the Pt metal, the oxide can be readily regenerated via redox reaction with water.^62,63^ Nb_2_O_5_ is a well-known reducible oxide with high oxygen storage capacity.^28^ Furthermore, the redox property of Nb_2_O_5_ under anodic electrochemical conditions in an acidic solution is well established.^28^ Therefore, Nb_2_O_5_ can facilitate the oxidation of adsorbed CO at lower potentials, which is described by the bifunctional mechanism represented in eqs 4–6.

4 $$Pt+{CH}_{3}OH\to {Pt-CO}_{ads}+{4H}^{+}+{4e}^{-}$$

5 $${Nb}_{2}O_{5}+H_{2}O\to {Nb}_{2}O_{5}{-OH}_{ads}+H^{+}+e^{-}$$

6 $${Pt-CO}_{ads}+{Nb}_{2}O_{5}{-OH}_{ads}\to Pt+{Nb}_{2}O_{5}+{CO}_{2}+H^{+}+e^{-}$$

Methanol preferentially binds to Pt atoms and undergoes dehydrogenation to form CO_ads_ intermediates, which are the main poisoning species for Pt atoms (eq 4). At low potentials, OH_ads_ species can be generated by water dissociation at Nb_2_O_5_ sites through the reaction described in eq 5. The CO_ads_ on Pt is continuously oxidized to CO_2_ with neighboring Nb_2_O_5_–OH_ads_ (eq 6), resulting in reduced CO poisoning. Nb_2_O_5_ can readily donate surface oxygen to Pt–CO_ads_ via a spillover mechanism.^61,64^ Oxygen species on the Nb_2_O_5_ surface can promote the oxidation of adsorbed CO on Pt active sites to CO_2_ at lower potentials. Therefore, it can be concluded that the combined interaction between Pt and Nb (oxides) in the present catalytic system plays a fundamental role in enhancing CO oxidation.

To allow a fair comparison of catalytic performance, both specific activity and mass activity were evaluated (Figure 6). The specific activity, obtained by normalizing the peak current to the ECSA, reflects the intrinsic efficiency of the active sites, while the mass activity, calculated by normalizing the current to the Pt mass on the electrode, provides an indicator of catalyst utilization and economic efficiency. The mass activity increased from 307 mA mg_Pt_^–1^ for Pt/C to 500 mA mg_Pt_^–1^ for Pt_3_Nb_1_/C, while the specific activity increased from 0.59 mA cm^–2^ to 0.79 mA cm^–2^. These results clearly demonstrate that the promotional effect of Nb_*x*_O_*y*_ in the catalyst system significantly enhances methanol oxidation activity, surpassing that of conventional Pt/C catalysts.

**Figure 6:**
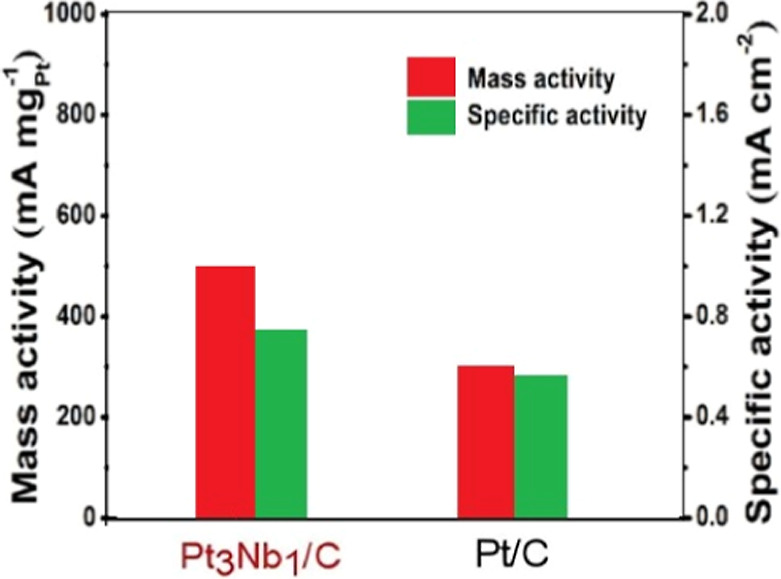
Mass activity, specific activity of Pt/C and Pt_3_Nb_1_/C catalysts at the forward peak potential for methanol oxidation in 0.5 mol L^–1^ H_2_SO_4_ with 0.5 mol L^–1^ CH_3_OH solution.

The evaluation of the electrocatalytic performance of materials for MOR requires careful analysis of the kinetic parameters, including current density, onset potential, and rate constants. These parameters are influenced by various factors, including the catalyst composition, particle morphology, conductive support, and the electrochemical conditions employed. Pt-based catalysts modified with promoter elements such as tin (PtSn) and ruthenium (PtRu) have been extensively studied due to their ability to enhance activity and improve tolerance to adsorbed intermediates, such as CO.^10,11^ Therefore, a direct comparison between the results obtained in this study and previously reported data enables the positioning of the developed materials’ performance within the context of current research. Table 5 presents a comparison of some MOR parameters in acidic medium, as determined in this study, with those reported in the literature for similar systems, such as PtSn and PtRu, enabling a critical evaluation of the observed electrocatalytic performance.

**Table 5:** Comparison of Kinetic Parameters for MOR of Pt-Based Electrocatalysts Supported on Vulcan Carbon Obtained Using CV in 0.5 M H_2_SO_4_ at 20 mV s^–1^

catalyst	metal ratio	metal loading (wt %)	methanol conc. (mol L^–1^)	ECSA (m^2^ g^–1^)	*I*_f_/*I*_b_	onset potential (V)	source
Pt/C	-	20	0.5	51.8	1.11	0.55	this work
Pt_3_Nb_1_/C	3:1	28	0.5	63.2	0.94	0.45	this work
Pt_3_Sn_1_/C	3:1	24	0.5	63.0	2.09	0.35	^10^
Pt_2_Ru_1_/C	2:1	60	2.0	72.4	1.30	0.50	^11^

It is evident that incorporating a second metal has a substantial impact on catalytic activity, CO tolerance, and overall performance. On the other hand, the monometallic Pt/C catalyst exhibited a moderate *I*_f_/*I*_b_ ratio of 1.11, indicating acceptable CO tolerance. However, its higher onset potential (0.55 V) suggests lower catalytic activity at low overpotentials, limiting its efficiency for practical applications. In contrast, Pt_3_Nb_1_/C showed a lower onset potential (0.45 V) and relatively high electrochemical surface area (ECSA = 63.2 m^2^ g^–1^), indicating improved surface availability for reaction. The *I*_f_/*I*_b_ ratio of 0.94 reflects a moderate ability to oxidize carbonaceous intermediates. The improved performance is attributed to the oxophilic nature of Nb, which can promote the formation of surface oxygen species that assist in CO oxidation.^28^

Pt_3_Sn_1_/C demonstrated the lowest onset potential (0.35 V) among the catalysts compared in Table 5, confirming excellent activity under low-potential conditions. Remarkably, it achieved the highest *I*_f_/*I*_b_ ratio (2.09), indicating superior resistance to CO poisoning and enhanced methanol oxidation to CO_2_. These effects can be associated with both bifunctional mechanisms and electronic modifications induced by Sn addition.^10,65^

The Pt_2_Ru_1_/C catalyst presents the highest ECSA (72.4 m^2^ g^–1^) and a high *I*_f_/*I*_b_ ratio (1.30), along with a favorable onset potential of 0.50 V, reflecting good overall performance. The well-established bifunctional role of Ru in providing OH species at lower potentials enhances CO oxidation, making Pt_2_Ru_1_/C one of the most efficient catalysts for DMFCs.^11^

Overall, all three bimetallic catalysts outperform Pt/C, confirming the beneficial effects of alloying Pt with oxophilic elements such as Nb, Sn, and Ru. Considering the experimental conditions employed, including methanol concentration, the electrolyte used, electrochemically active surface area, and scan rate, the results indicate that the investigated material (Pt_3_Nb_1_/C) exhibits competitive performance toward MOR under the specific conditions of this work.

Catalyst inks were prepared using equal masses of each catalyst, which were subsequently deposited onto the working electrodes under identical conditions. Under these standardized loading conditions, the Pt_3_Nb_1_/C catalyst exhibited superior electrocatalytic activity compared to Pt/C. Thus, incorporating Nb enhanced the catalyst’s MOR activity, exerting positive effects at both the electronic and morphological levels.

### 3.5 Chronoamperometry

Figure 7 shows the chronoamperometry (CA) profiles obtained for the commercial Pt/C catalyst and the Nb-doped material, Pt_3_Nb_1_/C, during 1800 s of polarization at 0.55 V vs RHE in a methanol-containing medium.

**Figure 7:**
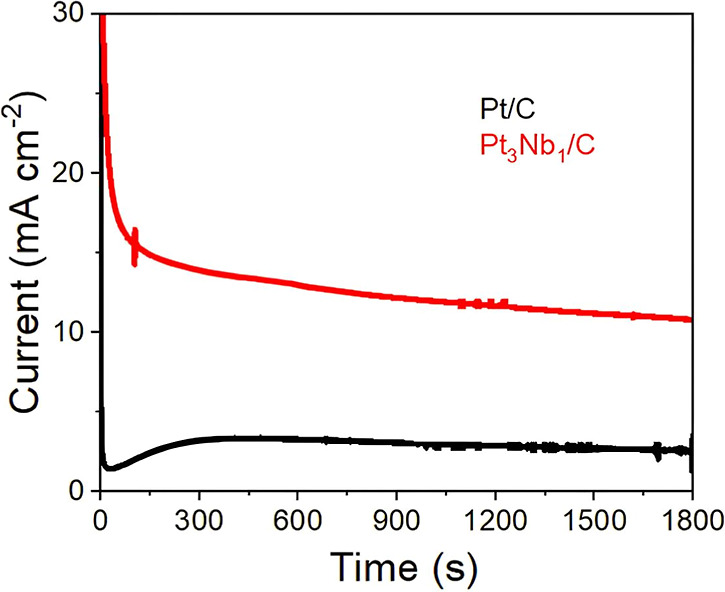
Chronoamperometry curves for commercial Pt/C and Pt_3_Nb_1_/C electrocatalysts at 0.55 V vs RHE in 0.5 mol L^–1^ H_2_SO_4_ + 0.5 mol L^–1^ CH_3_OH at 25 °C.

CA measurements were performed for 30 min (1800 s) to evaluate short-term catalytic stability and resistance to poisoning during methanol oxidation under acidic conditions (0.5 mol L^–1^ H_2_SO_4_). This approach is used in the literature as a standard initial assessment of catalyst stability and the effects of surface poisoning on the MOR. In fact, studies investigating Pt-based electrocatalysts report chronoamperometric stability tests in similar time windows (typically 20–50 min) to compare catalytic behavior and poisoning tolerance under controlled electrochemical conditions.^66,67^

A significant difference in current density is observed between the two systems, highlighting the superior electrocatalytic performance of the Nb-containing material. In the initial seconds of the measurement, both catalysts exhibit a sharp drop in current density, typical of the formation of adsorbed intermediates such as CO. However, the Pt_3_Nb_1_/C catalyst maintains a current density approximately three times higher over time than the commercial Pt/C. This behavior indicates greater electrochemical stability and resistance to catalytic deactivation, crucial factors for practical applications. However, it should be noted that the 30 min chronoamperometric test provides only a preliminary assessment and does not capture long-term degradation mechanisms, such as Nb_2_O_5_ dissolution or Pt sintering at high potentials. These results are nevertheless in agreement with recent studies that emphasize the incorporation of Nb oxides as an effective strategy to improve the activity and stability of Pt-based catalysts.^68,69^ Black-Araujo et al.^68^ reported significantly superior performance for Pt supported on silicon-doped niobium suboxide (Pt/NbOS) electrocatalysts, attributing the improvements to the formation of oxygenated species at lower potentials that facilitate the removal of adsorbed CO. Similarly, Hu et al.^69^ observed a mass activity about 12 times higher for PtNi/Nb_2_O_5_–C catalysts compared to commercial Pt/C, with enhancements attributed to the electronic combined interaction between Pt, Ni, and Nb_2_O_5_. Therefore, the data presented in this section reinforce the effectiveness of Nb doping in improving the performance of Pt-based catalysts for MOR, aligning with current trends in the research of new materials for clean energy applications.

The catalyst with a Pt/Nb atomic ratio of 3:1 was employed as the starting point to investigate the effects of Nb incorporation into commercial Pt/C systems. Structural and electrochemical analyses indicate that Nb doping can induce morphological changes at the nanoscale and potentially enhance catalytic activity under relevant electrochemical conditions. This composition serves as the baseline for a broader investigation to optimize the Nb content and understand the interaction mechanisms between Pt and Nb species. Future studies will focus on varying Nb concentrations, correlating physicochemical modifications with catalytic behavior, and evaluating long-term stability and activity toward target electrochemical reactions. These findings lay the groundwork for the rational design of advanced Pt-based electrocatalysts with improved performance for fuel cell and related energy conversion applications.

## 4 Conclusions

The study demonstrated that niobium (Nb) doping in the Pt_3_Nb_1_/C catalyst results in superior catalytic performance compared to the commercial Pt/C catalyst. The presence of Nb did not alter the crystalline structure of Pt, indicating that this metal is present either as an oxide (Nb_*x*_O_*y*_) or in an amorphous phase. HRTEM analysis revealed that the Pt_3_Nb_1_/C particles exhibited larger average diameters (3.10 ± 0.38 nm) than those of Pt/C (1.88 ± 0.20 nm), along with a higher specific surface area. The Nb-doped catalyst showed a predominance of metallic Pt species, whereas the commercial catalyst presented a higher proportion of Pt­(II) oxides. Changes in binding energies indicate a significant electronic interaction between Pt species and Nb_*x*_O_*y*_, supporting the hypothesis of combined catalytic effects. The electrochemically active surface area of Pt_3_Nb_1_/C (63.2 m^2^ g^–1^), compared to Pt/C (51.8 m^2^ g^–1^), indicates greater efficiency for Nb-doped Pt particles. The onset potential for methanol oxidation was lower for Pt_3_Nb_1_/C (0.45 V) than for Pt/C (0.55 V), indicating higher catalytic efficiency and, consequently, lower CO poisoning. It should be noted that the present study focuses primarily on the synthesis, structural characterization, and initial electrocatalytic performance of the Pt_3_Nb_1_/C catalyst toward methanol oxidation. Although the electrochemical measurements provide relevant insight into the catalytic behavior and indicate improved activity and tolerance to poisoning compared to Pt/C, a comprehensive assessment of long-term durability and structural evolution under extended electrochemical operation was beyond the scope of this work. In particular, systematic investigations involving prolonged cycling tests and postoperational structural characterization would provide valuable information regarding catalyst stability and possible structural changes during operation. Such studies represent an important direction for future research aimed at further elucidating the durability and mechanistic aspects of Nb-modified Pt electrocatalysts for alcohol oxidation reactions. The results demonstrate that the combined interaction between Pt and Nb oxides facilitates the removal of adsorbed CO, promoting its oxidation to CO_2_ at lower potentials. Moreover, the higher oxidation current density observed for Pt_3_Nb_1_/C further supports the positive impact of Nb doping on catalytic activity.
